# Current concepts in the pathogenesis of periodontitis: from symbiosis to dysbiosis

**DOI:** 10.1080/20002297.2023.2197779

**Published:** 2023-04-02

**Authors:** Ali A. Abdulkareem, Firas B. Al-Taweel, Ali J.B. Al-Sharqi, Sarhang S. Gul, Aram Sha, Iain L.C. Chapple

**Affiliations:** a Department of Periodontics, College of Dentistry, University of Baghdad, Baghdad, Iraq; b College of Dentistry, University of Sulaimani, Sulaimani, Iraq; c Periodontal Research Group, Institute of Clinical Sciences, College of Medical & Dental Sciences, University of Birmingham, Birmingham, UK

**Keywords:** Dental biofilm, symbiosis, dysbiosis, inflammation, periodontal disease

## Abstract

The primary etiological agent for the initiation and progression of periodontal disease is the dental plaque biofilm which is an organized aggregation of microorganisms residing within a complex intercellular matrix. The non-specific plaque hypothesis was the first attempt to explain the role of the dental biofilm in the pathogenesis of periodontal diseases. However, the introduction of sophisticated diagnostic and laboratory assays has led to the realisation that the development of periodontitis requires more than a mere increase in the biomass of dental plaque. Indeed, multispecies biofilms exhibit complex interactions between the bacteria and the host. In addition, not all resident microorganisms within the biofilm are pathogenic, since beneficial bacteria exist that serve to maintain a symbiotic relationship between the plaque microbiome and the host’s immune-inflammatory response, preventing the emergence of pathogenic microorganisms and the development of dysbiosis. This review aims to highlight the development and structure of the dental plaque biofilm and to explore current literature on the transition from a healthy (symbiotic) to a diseased (dysbiotic) biofilm in periodontitis and the associated immune-inflammatory responses that drive periodontal tissue destruction and form mechanistic pathways that impact other systemic non-communicable diseases.

## Introduction

Periodontal disease is a broad term used to encompass diseases and conditions of the periodontal tissues. The two major forms induced by dental plaque biofilm accumulation are gingivitis and periodontitis. Gingivitis is an inflammatory lesion that remains confined to the gingiva, but which may, in susceptible people, progress to a more severe and destructive form, periodontitis [1]]. A causal relationship between periodontitis and systemic diseases has not yet been robustly established, however studies indicate that periodontal pathogens and consequent immune-inflammatory responses to them are independently associated with the pathogenesis of several systemic diseases such as diabetes mellitus, atherosclerotic cardiovascular diseases, chronic obstructive pulmonary diseases, Alzheimer’s, chronic kidney disease, rheumatoid arthritis and certain cancers [2–5]. The ulcerated pocket epithelium provides a direct portal of vascular entry for periodontal pathogens, e.g. *Porphyromonas gingivalis*, *Aggregatibacter actinomycetemcomitans*, *Tannerella forsythia*, *Eikenella corrodens*, and *Fusobacterium nucleatum* to the systemic circulation, which may directly or indirectly affect other organ systems [6–8]. A report issued in 2018 estimated the economic burden arising due to periodontal diseases as approximately $154.06 billion in the US and €158.64 billion in Europe [9].

The dental plaque biofilm, alongside other environmental, lifestyle and genetic risk factors, is the main etiological agent responsible for the development and progression of periodontitis. However, since the late 19^th^ century, understanding of this concept has undergone significant advancement, sometimes with conflicting evidence. The specific plaque hypothesis (SPH) emerged which attributed dental caries to specific bacteria in the dental plaque biofilm, mainly *Streptococcus mutans, S. sobrinus* and *lactobacilli* [10]. The latter bacteria were observed to be partly indigenous in nature and present in both health and disease, together with the recognition of other bacteria as potential periodontal pathogens which underpinned the SPH. This led researchers to propose the non-specific plaque hypothesis which stated that it was the sheer biomass quantity rather than specific microorganisms that were responsible for the development of periodontitis [11]. In 1994, Marsh suggested that ecological stress was the driver for imbalance in the oral microbiota, encouraging the outgrowth of pathogenic bacteria; this was known as the ecological plaque hypothesis (EPH) [12]. In the late 1990s, pioneering work by Socransky and co-workers categorized the periodontal bacteria according to their pathogenicity by assigning distinct color-codes to clusters that mapped to various states of health and disease [13]. This work, with the EPH, set the basis to the keystone-pathogen hypothesis, proposed by Hajishengallis and colleagues. This hypothesis explained the shifting of the microbiome from a symbiotic one to a biofilm characterized by dysbiosis induced and aggravated by low abundant ‘keystone pathogens’. For example, *P. gingivalis* elicits an intense/destructive host immune response [14]. The aim of this narrative review was to report the current literature on the transition from a health-promoting to a diseased-forming in periodontitis and the associated immune-inflammatory response.

## Formation and development of the dental plaque biofilm

### Formation of acquired pellicle

**Figure 1.**
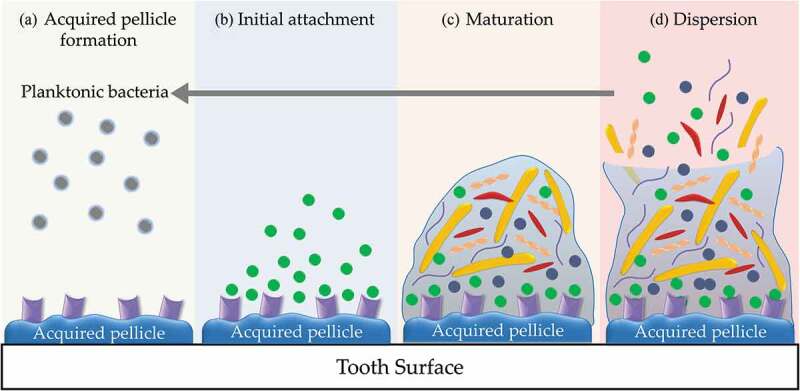
Biofilm formation and development in the oral cavity. a. acquired pellicle formation; b. initial attachment of early colonizers; c. maturation of biofilm and coaggregation of bacteria; D. dispersion of bacteria.

Colonisation of organisms within the dental biofilm should be preceded by the presence of a condensed layer of macromolecules at the base of the biofilm known as the acquired pellicle (Figure 1) [15–17]. This layer was thought to be derived principally from salivary glycoproteins; however, a recent study implicated an important contribution from gingival crevicular fluid (GCF) in the formation of this layer [18,19].

### Adhesion of bacteria

Planktonic bacteria in the oral cavity attach to specific pellicle-associated binding sites such as acidic proline-rich proteins and α-amylase for attachment of early colonizers (Figure 1) [20–22]. Adhesion of bacteria at this stage is mainly dependent upon weak bonds, e.g. Lifshitz-van der Waals, Lewis acid-base and electrostatic interactions [23,24]. The strength of this attachment is increased with the formation of extracellular polymeric matrix (EPM) [25,26]. In addition to the biofilm matrix, attachment of bacteria within the biofilm is mediated by specialized appendages called fimbriae or pili that are composed of subunits called fimbrillins, possessing adhesins that selectively adhere to pellicle-coated teeth or to other bacteria [27]. Fimbriae are common among many bacterial species including *Streptococci*, *Actinomyces* and *P. gingivalis* [28–30]. Furthermore, fibrils also facilitate bacterial attachment; these structures are shorter and different in morphology and distribution from fimbriae [31]. Fibrils can be found in some oral bacteria such as *Prevotella intermedia*, *P. nigrescens*, and some Streptococcal strains [28,29,32,33]. Moreover, motile Gram-negative bacteria can utilize force-generating motility as a mechanism for initial attachment to the tooth surface, which counteracts repulsion forces. This active or ‘twitching motility’ is attributed to flagella and type IV pili, respectively [27]. Notably, other surface proteins such as autolysin [34] and capsular polysaccharide [35] also play a role in the attachment of bacteria to solid surfaces.

### Maturation of biofilm and coaggregation of bacteria

Maturation of the dental plaque biofilm (Figure 1) starts with the recognition by late colonizers including *F. nucleatum*, *Treponema denticola*, *T. forsythia*, *P. gingivalis*, *P. intermedia* and *A. actinomycetemcomitans* of polysaccharide or protein-binding sites on the cell surface of primary colonizers [36,37]. Consequently, the relative number of late colonizers in the dental plaque biofilm increases at the expense of primary colonizing bacteria such as *Streptococci* and *Neisseria* [38,39]. The tendency of paired-aggregation has been shown in 90% of dental biofilm-associated bacteria [40]. However, this coaggregation is not random, and receptor sites of each bacterial species have specificity for complementary binding to the adhesion molecules of certain bacteria. For instance, coaggregation occurs between *F. nucleatum* and *S. mutans*; yet, the latter lack the ability to bind to *P. gingivalis* [23]. Additionally, coaggregation bridges are another feature of certain bacterial species which possess different receptor sites capable of binding to two or more other bacterial species. *F. nucleatum* is the most well-known example of a bridging species, which mediate coaggregation of aerobic and strictly anaerobic bacteria [23]. Coaggregations of bacteria in a mature dental biofilm exhibit unique patterns, e.g. ‘corn on the cob’ structures and ‘bristle brush formations’ [36,37].

**Figure 2.**
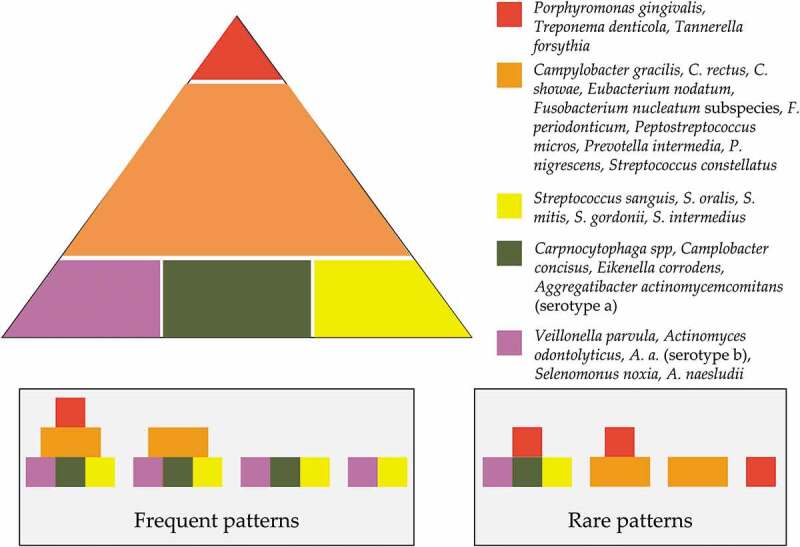
The association among subgingival species (adapted from Socransky et al. [13,27]). Presence of 40 subgingival species and the association among them in subgingival dental biofilm samples (*n* = 13,321) were analysed using checkerboard DNA-DNA hybridization and cluster analysis and community ordination techniques, respectively. The base of the pyramid represents the early colonizers, followed by the orange complex, which bridges the early colonizers with the red complex that dominates the biofilm at the advanced stages of periodontitis.

The work of Socransky et al. provided an in-depth understanding of the biofilm-associated bacterial communities of the subgingival microbiota [13]. The bacteria were divided into five complexes (Figure 2) which received a unique color coding [41]. *P. gingivalis*, *T. forsythia* and *T. denticola* belong to the red complex of pathogens and are mainly found in subjects with periodontitis. Additionally, the orange complex pathogens, *Fusobacterium*, *Prevotella*, and *Campylobacter* species, are often related to periodontitis. The other complexes such as the yellow complex (composed of different *Streptococcus* species) and green complex (composed of *Capnocytophaga* species) were strongly associated with periodontal health. Yellow, green, and purple complexes are the primary colonizers and considered as a prerequisite for the appearance of the orange and red complexes (secondary colonizers). Although this pattern of coaggregation was the most frequent (Figure 2), rarely, certain complexes could be detected in the absence of other bacteria [41].

### Dispersion of bacteria

With the increase in the mass and bacterial populations of the dental biofilm, solitary bacteria or bacterial clusters become detached and return to a planktonic state (Figure 1) [42]. There remains no consensus about a single dispersal mechanism of bacteria; however, two mechanisms were proposed, namely active and passive dispersion [43–45]. Active dispersion is attributed to the bacteria themselves, while passive dispersal is provoked by external forces such as shear forces from salivary flow, competition with other bacteria, and mechanical debridement [42]. In general, dispersion of bacteria occurs by three mechanisms: erosion, sloughing and seeding [46,47]. While erosion and sloughing may be active or passive, seeding is an active process limited to hollow cavities within the biofilm from which large numbers of solitary cells or bacterial masses are rapidly detached [48,49].

## Structure of the dental biofilm

**Figure 3.**
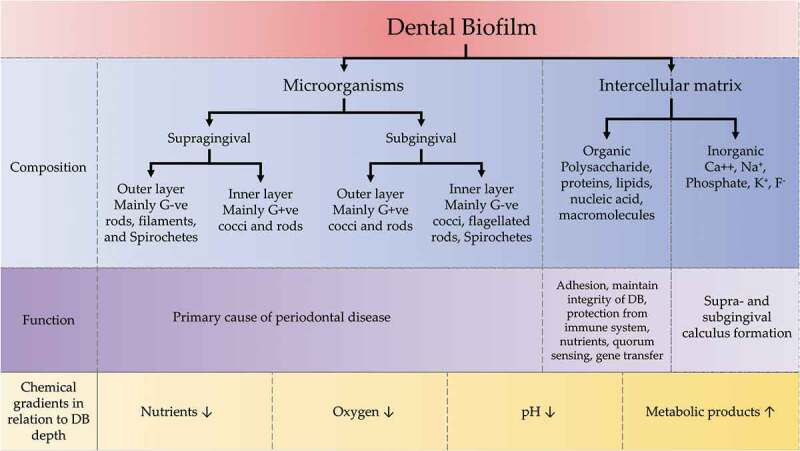
Components of dental biofilm with their functions and relation of chemical gradients to the depth of dental biofilm. DB: dental biofilm, G+ve: Gram-positive, G-ve; Gram-negative.

Biofilms are composed of microbial cells enclosed within EPM which is derived from inhabitant microbiota and the host [50,51]. Molecular-based sequencing studies revealed that about 700 species contribute to the bacterial component of dental plaque biofilms [52]. The microorganisms within biofilm are not haphazardly arranged but functionally and spatially organized [53]. The type of microbiota and the load of bacterial species detected in the dental biofilm varies according to their location or niche [52,54]. The number of bacteria in the supragingival biofilm on an individual tooth surface can exceed 10^9^ cells. In a periodontal pocket, counts can range from 10^3^ bacteria in a healthy crevice to >10^8^ bacteria in a deep pocket [55,56] (Figure 3).

Mature biofilms exhibit shared structural characteristics such as microcolonies of bacterial cells and water channels [57]. Furthermore, steep chemical gradients exist within dental plaque biofilm. Towards the deeper part of the biofilm, it has been reported that nutrients, O_2_ and pH are lower in concentration, whereas metabolic products are highly prevalent [58–60]. The EPM of dental plaque biofilms consists of inorganic and organic materials derived from the host, mainly saliva, GCF and bacterial products [54]. The inorganic materials include mainly calcium and phosphate ions, as well as small amounts of other minerals such as sodium, potassium and fluoride, which play a critical role in the formation of dental calculus [61]. While the organic components are integral to bacterial attachment, protecting individual bacteria from the immune system, providing nutrients, quorum sensing, and facilitating horizontal gene transfer [50] (Figure 3).

## Microbial virulence and metabolism

**Figure 4.**
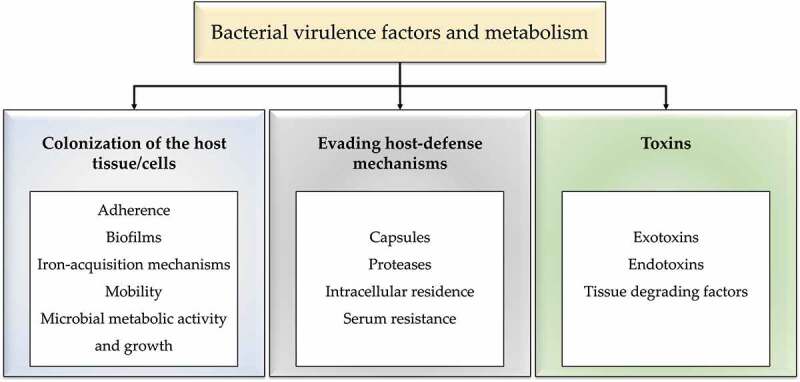
Bacterial virulence factors and metabolism.

Virulence factors are those characteristics that allow a microorganism to cause disease by evading or subverting the immune response and other host defence systems. Virulence factors generally comprise three essential functions necessary for bacterial surface colonization of host tissues/cells (Figure 4): evading host-defense mechanisms and initiating disease symptoms (toxins) [62].

### Colonization of the host tissues/cells

#### Adherence

As previously discussed, the adhesive properties of fimbriae are integral to biofilm formation by mediating the coaggregation of different bacterial species together and facilitating their attachment to the hosts’ tissues [63]. For example, *P. gingivalis*-fimbriae mediate aggregation with the surface protease of *T. denticola* [64–66] and human glyceraldehyde-3-phosphate dehydrogenase, facilitating the invasion of this bacterium into the host cells [63,67].

#### Biofilms

The EPM of the biofilm is mainly composed of macromolecules that, in addition to their protective role, maintain a close proximity between bacterial and host cells. This sustained host-bacterial interaction either results in promoting health or causing disease [50]. In addition, matrix macromolecules such as adhesins and extracellular DNA participate in the initial attachment to salivary pellicle and to coaggregation of bacteria within the biofilm [68,69].

#### Iron acquisition mechanisms

The bulk of iron in the body is found in intracellular complexes; however, the amount of free ionic iron is insufficient to support the growth of bacteria [62]. Pathogenic bacteria have developed several mechanisms to acquire iron from the microenvironments [70]. These heme-acquisition pathways include hemagglutinins, hemolysins, gingipains, different outer membrane receptors (HmuR, IhtA and HusB), siderophore/hemophore-like proteins (HmuY and HusA) and intracellular heme transport, storage and processing, e.g. PgDps [71]. Gaining access to iron supply by the putative periodontal pathogens supports their growth, thereby contributing to shifts in microbial ecology and an emergent dysbiosis of the biofilm [72].

#### Motility

Mobility is an evolutionary mechanism that together with chemotaxis enables bacteria to seek nutrients and find favorable niches for colonization [73]. The flagella-assisted motility is the most common molecular motor utilized by periodontal pathogens including spirochetes. The flagella-mediated motility and chemotaxis-mediated motility are also considered as virulence factors [74] and the extent as well as the degree of motility differ among different bacterial species. Furthermore, the location of spirochetal flagella varies, e.g. *T. denticola* is in the periplasmic space, while other bacteria exhibit exposed flagella [75]. This unique periplasmic localization enhances motility in highly viscous microenvironments, evading the immune system, and provides protection from antibodies secreted by the host specifically against flagella proteins [76,77].

#### Microbial metabolic activity and growth

Increased growth is another way in which periodontal bacteria can evade natural removal and mechanical debridement. The periodontal pocket is an appropriate habitat for anaerobic, fastidious, low adherent, motile bacteria such as the red and orange complexes [78,79]. Additionally, the subgingival domain provides mechanical protection, anaerobiosis, and nutrients to the periodontal bacteria, including particular growth factors such as hemin, vitamins, and hormones, as well as serum components from GCF [80].

Proteins, peptides, and amino acids are the key nutrient sources in GCF, and they are targets for the highly proteolytic red and orange complex bacteria. The key variables that enable the simultaneous development and increase in the inflammatory reaction are increased subgingival bacterial growth and altered ecology, i.e. dysbiosis. Short-chain carboxylic acids (butyric acid, propionic acid, valeric acid, capronic acid, and phenyl acetic acid), ammonia, and hydrogen sulfide are hazardous metabolites produced by increased metabolic activity [79]. Subsequent multiplication of the periodontal bacteria leads to a rise in metabolic activity, bacterial number and adaptation of new bacterial species results in a constantly changing ecology that lasts until the bacterial community and the host reach homeostasis. Such equilibrium may persist for long periods, or even lifelong for low-periodontitis susceptible individuals (Figure 4) [79].

### Evading host-defense mechanisms

#### Capsules

Many pathogenic microorganisms, mainly Gram-negative anaerobic rods, produce a capsule e.g. *P. intermedia* and *P. gingivalis*. The capsule serves as an anti-phagocytosis mechanism for neutrophils and macrophages. In addition, encapsulated strains possess higher virulence potential than non-capsulated bacteria by modulating the immune system and supporting survival of bacteria within cells [81,82]. However, the *in vivo* role of the capsule in the pathogenicity of encapsulated bacteria is not well elucidated, as most of the evidence is derived from experimental animal models [82].

#### Proteases

*P. gingivalis*-gingipains differentially affect neutrophil chemotaxis and activation by cleaving IL-8 to a more active-chemokine form; hence, enhancing recruitment PMNLs which contribute to periodontal tissue destruction [83]. The outer membrane vesicles released by *P. gingivalis* into the microenvironment are also capable of destroying IL-8, offering notable protection from host defenses [84]. In addition, *P. gingivalis*-gingipains have the ability to attack and degrade C3 which gives the invading bacteria a substantial survival advantage [85].

#### Intracellular residence

It is well known that periodontal pathogens remarkably evolved to evade the immune system; one such method is by intracellular invasion of the host’s cells. Advanced microscopic techniques revealed that periodontal pathogens including *P. gingivalis*, *T. forsythia, A. actinomycetemcomitans, F. nucleatum, P. intermedia*, and *T. denticola* were collectively located in the buccal epithelial cells [86]. Lee et al., (2018) demonstrated a novel mechanism of *P. gingivalis* for intracellular invasion of gingival epithelial cells by exploiting the autophagy machinery of these cells [87]. Similarly, for *T. denticola*, intracellular localization is the most predominant pattern associated with utilizing cellular machinery to over-produce chymotrypsin-like proteinase responsible for tissue destruction in periodontitis [88]. *A. actinomycetemcomitans* is another bacterium which invades gingival epithelial cells mainly by inducing F-actin rearrangement via FAK-signaling downstream [89].

#### Serum resistance

Pathogens that cross mucosal or skin barriers but persist in the extracellular environment almost always require protection from complement-mediated lysis. The lytic impact of serum on Gram-negative bacteria is mediated by complement and can be triggered by either the classical or alternative pathways (Figure 4) [90].

### Toxins

Bacterial toxins are among the classical virulence factors that have been linked with bacterial infections [91]. Most toxins are highly specific proteins that have evolved to influence certain components of host cell signalling for the bacteria’s benefit (Figure 4) [92]. Leukotoxin is an example of an exotoxin that is dedicated to damage leukocytes, and which can be found in several pathogenic bacteria, such as *A. actinomycetemcomitans*, the PV-leucocidin in *S. aureus* [93] and *F. necrophorum* [94,95]. LPS is the endotoxin of Gram-negative bacteria, containing a toxic lipid portion (lipid A) that is incorporated into the outer membrane responsible for the release of cytokines and activation of the complement and coagulation cascades [90].

Bacterial tissue-degrading or histolytic enzymes are directly responsible for periodontal tissue destruction. For example, hyaluronidase ‘spreading factor’, chondroitin sulphatase and beta-glucuronidase released by *Streptococci*, *Peptostreptococci* and *Corynebacteria*. Similarly, different bacterial proteases, such as collagenases and *P. gingivalis*-gingipains are known to play a crucial role in degrading tissue components during infections [81] and degrade host defense molecules such as immunoglobulins and complement [96].

## Oral microbiota from birth to adulthood

At parturition, the microorganism-free intrauterine environment transitions to the extra-uterine environment with exposure to microorganisms [97]. This may also be influenced by the mode of birth, with a more diverse oral microbiota reported in vaginally born children compared to those born by caesarian section [98], which may also lower exposure of the new born to the protective commensal bacteria acquired from the mother at birth [99]. For instance, higher numbers of taxa detected in three-month-old infants delivered vaginally relative to C-section, which was exclusively associated with a high prevalence of a novel species of *Slackia exigua*, which has also been isolated from the subgingival biofilm of periodontitis-involved teeth [100].

The evolution of microbial communities continues within the first months of an infant's life, reaching a distinct oral microbiota derived from the mother by 5 months of life [101]. However, a significant shift in the microbial composition of the infants’ oral cavities arises following the eruption of the first tooth/teeth. The introduction of a non-shedding tooth surface within the oral cavity creates a novel ecological niche. This stage has previously been described as ‘window of infectivity’ [102], with the principal role of *S. mutans*, a cariogenic species on teeth, however *S. mutans* has also been described in edentulous children, suggesting a potential role of the soft tissues as a reservoir for this pathogenic species [103]. At 3 years of age, the oral microbiome becomes more complex and continues to mature through the various stages of tooth development, from primary, early mixed, late mixed, and the permanent dentition. In parallel with this, a higher prevalence of *Pseudomonaceae*, *Enterobacteriaceae* and *Pasteurellaceae* (genus *Aggregatibacter*), and *Moraxellaceae* (genera *Acinetobacter* and *Moraxella*) species have been reported within primary dentition. As the dentition transitions from deciduous to permanent, the prevalence of *Veillonellaceae* and *Prevotella* increase, whilst that of the *Carnobacteriaceae* family decreases [104]. In addition, the increased proportions of Bacteroidetes (mainly the *Prevotella* genus), and Spirochaetes were detected with increasing age [104], with a significant abundance reported in the adult population [105]. In addition, puberty is characterized by hormonal changes that generate nutritional enrichment within oral environment, resulting in increases in Gram-negative anaerobes and spirochetes [106].

Notably, the adult oral cavity is host to different microbiomes throughout life, which are unique for every oral niche and which also act as continuum that dynamically interact with each other. Recent evidence suggests that periodontitis is not only driven by the subgingival biofilm but by microbiomes from other niches such as the dorsum of the tongue [107]. In addition, Stephen et al. (2021) demonstrated that the tongue may act as a reservoir for subgingival periodontal pathogens, e.g. *Gemella morbillorum*, *T. denticola* and *Peptostreptococcus stomatis* responsible for increasing bleeding scores and the percentage of deep periodontal pockets [108]. Furthermore, the severity of periodontitis was strongly associated with the total bacterial count, and also with *Archaea* on the tongue [109]. Patients with acute tonsillitis exhibited an abundance of periodontal pathogens such as *Prevotella*, and *Fusobacterium* species [110], and the prevalence of periodontitis increased in patients with peritonsillar disease and recurrent tonsillitis [111]. However, the composition of the subgingival biofilm did not change following tonsillectomy [112], a finding supported by results from another cohort study, which demonstrated that tonsillectomy did not reduce the risk of periodontitis [113]. These apparently conflicting results may be attributed to several environmental factors that directly influence the bacterial composition of the oral cavity such as the type of food, smoking, consumption of medications, individual immune responses [114], and different sampling techniques [115].

## Dental biofilm: the shift from health to disease

The process of transition from periodontal health to the advanced stages of periodontitis is associated with a microbial shift from the major symbiotic bacteria known as ‘symbionts’ to dysbiosis, with high proportions of pathogenic bacteria, the so-called ‘pathobionts’. This transition is influenced by several stressors, including the host immune-inflammatory response, individual susceptibility, and behavioural risk factors such as smoking.

At an early stage, the hosts’ inflammatory-immune response is induced following microbial biofilm accumulation at and below the gingival margin. This increases GCF flow which in turn delivers protective components of the innate immune response such as neutrophils, complement and cytokines [116], together with host molecules such as haemoglobin, which is used as a substrate for proteolytic bacteria such as *P. gingivalis* [117]. Although the innate response is effective against susceptible species, subversion of these responses by microbial tactics, such as subverting neutrophil function, affecting complement degradation, and inhibiting phagocytosis can be induced by others, e.g. *P. gingivalis* [118]. However, those species that are at risk may exploit the potential for cross-protection offered by neighbouring organisms within the biofilm structure, increasing their tolerance to the inflammatory response and also their survival.

The term ‘inflammophilic’ refers to those microbial consortia associated with periodontitis that can endure inflammation and use inflammatory conditions to survive and prosper [56], such as elevations in pH [119]. Following such local environmental changes, bacterial competitiveness and gene expression are altered and increased in different species such as *P. gingivalis* [120]. During inflammation, the volume and composition of GCF alters, forming a vital source of nutrients that effect continuous change in the microbial composition of the dental biofilm. To clarify the importance of such nutrients within GCF, several investigations have identified numerous nutritional inter-relationships between subgingival microbiota [121]. An example of this inter-bacterial dependency is the growth of *T. denticola* that is stimulated by isobutyric acid produced by *P. gingivalis*, whilst *T. denticola* produces succinic acid, that supports the growth of *P. gingivalis* [122]. This polymicrobial synergism within the dental plaque biofilm not only exists at the nutritional level but inter-microbial metabolic product and gene expression exchanges are necessary to sufficiently increase biomass virulence and induce disease, which cannot be triggered by weakly virulent individual species alone. This is consistent with the most cotemporary plaque hypothesis, which proposes a disproportionate dysbiotic influence of low abundance but virulent species on the whole microbial community, both directly and indirectly via host immune modulation/subversion [123,124]. However, the dysbiosis theory remains open to question, as a clear association between any of these putative pathogens to induce periodontitis in humans is lacking. This notion is supported by resistance to alveolar bone loss in a murine periodontitis model infected with *P. gingivalis* [125].

Meta-transcriptomic studies have confirmed the role of the entire microbiota to induce dysbiosis, rather than a limited number of putative pathogens [126]. In addition, several species that have been associated with health, including *Streptococci*, *V. parvula*, *P. fluorescence*, were highly active in transcribing virulence factors associated with other putative pathogens such as *P. gingivalis* and *T. forsythia*. Furthermore, the genes isolated from these sites were associated with cell motility, lipid A and peptidoglycan biosynthesis and transport of iron, potassium and amino acids. Several human microbiome studies have confirmed a pivotal role for the dysbiosis hypothesis in the pathogenesis of periodontitis. According to these studies: (i) health-associated microbiota differ from those related to periodontitis; (ii) the microbial diversity (phylotypes) in healthy subjects is lower than within periodontitis subjects; (iii) health-associated species are suppressed but not lost and (iv) there is merely a shift in the balance of species that dominate the subgingival environment rather than colonization by new microbial species [127].

Periodontal health-associated microbiota can remain in a state of stability over time and live in dynamic equilibrium with the host. The shift in microbial diversity from healthy gingiva in 1–2 weeks following oral hygiene cessation has been evaluated [128]. Bacteria that exhibited a negative correlation with bleeding on probing were mostly early colonizers, including aerobic and facultative anaerobic Gram-positive cocci and rods such as *Actinomyces, Rothia and Streptococcus*. As gingivitis developed in response to microbial biofilm accumulation, other species, mostly obligate anaerobes, with positive correlations with bleeding on probing were increased in number, such as *Campylobacter*, *Fusobacterium*, *Lautropia*, *Leptotrichia*, *Porphyromonas*, *Selenomonas*, and *Tannerella* species [129]. However, early gingivitis is still considered, in many ways, as a homeostatic condition and to represent stable inflammatory changes encountered by the host. Undoubtedly, bacteria represent the main aetiological agent to induce gingivitis; nevertheless, the host response against bacteria dictates whether disease will progress or not [130]. Indeed, uncontrolled or dysregulated host response patterns are the main drivers of tissue destruction [131].

Disease-associated microbiota constitute a minor component of the subgingival microbial community in health and increase considerably with the development of periodontitis [132]. In healthy periodontium (and in gingivitis), inter-microbial species competition seems to self-regulate, achieving microbial homeostasis. As inflammation proceeds and a pocket develops, the subgingival microbiome becomes dominated by Gram-negative anaerobes that exploit the local microenvironment, which is enriched with tissue breakdown products, plasma proteins, and various nutrients such as hemoglobin, and amino acids [12,133]. Thus, the microbial shift from health to disease is likely to involve a microbial succession process, in which the proportion of current species is altered by new colonizers [134].

It has been shown that all periodontitis-associated species can be detected in gingivitis and most were also detected in health [135]. In addition, depending on the severity of periodontitis, different ’clusters’ were present. For example, in a mild form of periodontitis, ‘cluster A’ was represented by species of the genera *Campylobacter*, *Corynebacterium*, *Fusobacterium*, *Leptotrichia*, *Prevotella*, *Tannerella*, and *Saccharibacteria*. Whereas in severe periodontitis, ‘Cluster B’ was enriched with ‘red complex’ bacteria, *Filifactor alocis*, *Treponema* species, and *Fretibacterium* species. The difference in the bacterial load of the same microbial species between periodontitis and healthy gingiva can be explained by the EPH [12], which attributes the microbial shifting process to pressures from the altered inflammatory stress that favours the growth of pathobionts.

Altered nutrient concentrations may support the outgrowth of proteolytic and asaccharolytic bacteria within the subgingival area via secretion of inflammatory exudate (GCF) and by haem-acquisition. This was supported by a transcriptomic study that revealed there was an increase in proteolysis, iron acquisition, peptide transport, and LPS synthesis-associated genes within subgingival biofilms, which could further promote the inflammatory potential of the associated bacteria [136]. Accordingly, the term ‘inflammophilic’ pathobionts has been applied to those subsets of species that may fail to endure the new environment, but that can further induce dysbiosis within the microbial community.

Another important inflammatory by-product that may promote dysbiosis is potassium ions, which were reported to be increased with increasing periodontal inflammation [126,137]. In addition, nitrate is considered another by-product that is increased during inflammation and further promotes dysbiosis by enhancing the anaerobic properties of *Enterobacteriaceae*. For instance, it has been found that the increased level of nitrate within the periodontal environment of *Gas6*^*-1-*^ mice, a deficiency in the growth arrest-specific gene 6, impacts by promoting microbial dysbiosis through selective expansion of nitrate reductase-expressing proteobacteria [138]. Undoubtedly, all of these metabolic and inflammatory by-products appear to be environmental cues, capable of remodelling the periodontal microbiota from a eubiotic into a dysbiotic one.

The cause-and-effect relationship between inflammation and dysbiosis has been the subject of debate [139,140] regarding whether inflammatory pressure can create dysbiosis by shifting the proportions of inflammophilic pathobionts and commensal symbionts, resulting in those dysbiosis-associated bacteria further exacerbating inflammation [126,138]. Therefore, it seems that neither the inflammatory process nor dysbiosis can be fully established without reciprocal interactions between these processes, creating a sustained circular loop that develops into periodontitis.

The crucial role of the hosts’ inflammatory response in changing microbial composition in the subgingival environment is important to recognise. It has been shown that 53 species were more abundant in healthy sites compared to 123 species found to be more abundant in periodontitis sites. This indicates that the immune subversion/modulation favours colonization of numerous symbiotic commensals that favour pathogen growth [141].

The microbial dysbiosis is not only influenced by inflammatory subversion and the local microbial environment but also by the genetic background of individual patients/people. For instance, subjects with leukocyte adhesion deficiency (LAD)-1 who have a genetic defect in CD18 or integrin-β chain-2 develop severe generalized periodontitis similar to those who have the aggressive type (currently known as grade C periodontitis in systemically healthy young adults) [142]. However, the microbial compositions/proportions in subjects with LAD-1 are different, with less complexity compared to those having the traditionally named chronic or aggressive forms of periodontitis [134,143,144]. Microbial species such as *A. actinomycetemcomitans*, which predominate in aggressive periodontitis patients, were undetectable or found in very low abundance in LAD-1 subjects. Conversely, species such as *Pseudomonas aeruginosa* [144,145] and *Leptotrichia* species that were not present in either chronic or aggressive periodontitis, were uniquely present in subjects with LAD-1. In addition, excessive production of interleukin (IL)-17 by T helper-17 cells in LAD-1 subjects [146] is likely to induce dysbiosis in the microbial biofilm.

**Figure 5.**
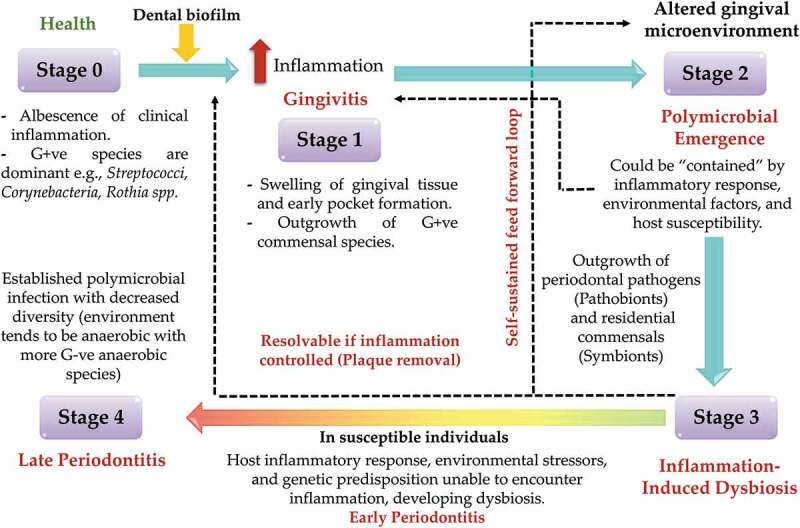
Inflammation-Mediated Polymicrobial Emergence and Dysbiotic Exacerbation (IMPEDE) model. According to this proposed model, plaque-induced periodontitis is mainly derived from inflammation. This model consists of 5 stages: stage 1: gingivitis, stage 2: emergence of polymicrobial diversity in early periodontitis, stage 3: inflammation mediated dysbiosis and opportunistic infection, and stage 4: late stage of periodontitis. Adapted from Van Dyke et al., 2020 [147].

In light of how dysbiosis and the associated aggravation of periodontal tissue destruction are driven by environmental changes, a new hypothesis, called the ‘Inflammation-mediated polymicrobial-emergence and disease exacerbation’ (IMPEDE) model [147] has emerged to complement the 2018 classification of periodontal diseases [148]. According to this classification, the continuity of periodontitis from health to disease is viewed through four stages of severity, complexity as well as extent and distribution. The IMPEDE model has emerged as a possible driver of the clinical conditions that could be manifest within each periodontitis stage. In a similar fashion to the clinical classification of periodontal disease, the IMPEDE model defines five stages encountered: health, gingivitis and periodontitis that may be developed, contained or progressed. In addition to the healthy state representing Stage 0 (absence of clinical inflammation), the other four stages represent disease development. Stage 1 is gingivitis, in which inflammation associated with the outgrowth of commensal bacteria in response to a non-specific buildup of dental plaque in susceptible individuals may cause gingival swelling and early pocket formation. Stage 2 is initial periodontitis, in which polymicrobial diversity is increased with increasing inflammation and dysbiosis is initiated. Stage 3 is inflammation-induced dysbiosis exacerbation via a self-sustained feedforward loop, the diversity is increased, with more pathobionts, and symbionts tending to favor the emergence of Gram-negative species. This is resolvable if the inflammation is controlled, allowing commensal microbiota to predominate again. Finally, Stage 4 is late periodontitis which in susceptible individuals is characterized by the emergence of polymicrobial infection with decreasing polymicrobial diversity; the associated environment is dominated by anaerobic microbial species and along with uncontrollable inflammation results in advanced tissue destruction (Figure 5).

## Inflammatory response of the host to the dental biofilm in periodontal disease

The relationship between a healthy symbiotic biofilm and the local host response remains in static equilibrium until environmental alterations drive alterations in the microbial ecology towards a destructive and dysbiotic microbiota. Maintaining a healthy state critically relies on the quantity, function and regulation of the host inflammatory cells that patrol the local tissues, being recruited at the target site and being involved in various innate and adaptive host responses.

The first-line host defence systems encountered by the microbial biofilm, supra- or subgingivally, include mucosal barriers, salivary defense mechanisms, PMN leucocytes, GCF and antimicrobial peptides [149]. In addition, bioactive lipids including resolvins, salivary mucins and agglutinins, IgA, IgG and activated complement with GCF may play a pivotal role as protective mediators to maintain a healthy periodontium [150,151]. Epithelial integrity acts as an important defensive physical barrier against microbial penetration during inflammation. Microbial-induced cytokines are released through direct interaction of the pattern recognition receptors (PRR) of the gingival epithelium such as Toll-like receptor (TLR), with Gram-positive and Gram-negative pathogen-associated molecular patterns (PAMPS) on the bacterial surface. This leads to the release of pro-inflammatory and regulatory cytokines such as IL-6, IL-8, and IL-1α, which stimulate epithelial cells to express antimicrobial peptides such as human β-defensins, calprotectin and cathelicidin.

The PMNs play a leading protective role against invading bacteria through their antimicrobial mediators, phagocytosis, degradative enzymes such as matrix metalloproteinases (MMP), or cytotoxic substances, e.g. reactive oxygen species [152,153]. The PMNs become prominent as an inflammatory trigger that continues through different stages of gingivitis and early periodontitis. However, the supremacy of PMNs is also evident during the burst model of periodontitis, in which there is acute exacerbation of periodontitis after a period of remission [154]. As the inflammation continues in response to microbial succession, in particular within the inflammophilic dysbiotic biofilm, the proteolytic trait of neutrophils becomes as dominant as its protective responses. This may lead to epithelial deformity that can provide a point of entry to microbial invasion into underlying connective tissues, which subsequently provokes tissue destruction and bone loss [155].

The destructive ability of neutrophils is magnified if their activities deviate from normal, due to excessive or diminished recruitment, dysfunction or hyperactivity, leading to exaggerated tissue breakdown [156]. In addition, the role of host genetics is key in maintaining host-microbial homeostasis. Single gene deficiencies such as deficiencies in the leukocyte function antigen, (LFA)-1 integrin, promoting neutrophil infiltration, and its antagonist, developmental endothelial locus (DEL)-1, which inhibits/regulates the neutrophil’s adherence to the endothelial wall and subsequent extravasation, have been shown to induce dysbiosis and bone loss in mice [123,157].

**Figure 6.**
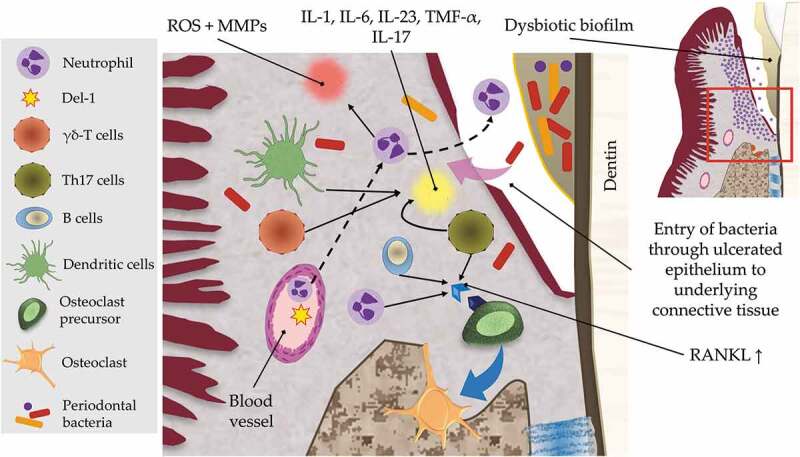
Neutrophils-induced inflammatory mechanisms involved in tissue destruction and bone loss. Neutrophils are recruited in a developmental endothelial locus (Del)-1-induced pathway into the gingival epithelium that fail to encounter the dysbiotic bacteria which invade the gingival connective tissue and interact with different host cells such as dendritic cells and γδ T cells. Host-bacterial interaction results in production of proinflammatory cytokines such as interleukin (IL)-1, IL-6, tumor necrosis factor (TNF)-α, IL-23, and IL-17. IL-17 has an activating influence on T helper (Th)-17 and B cells, which upon activation increase receptor activator of nuclear factor kappa-B ligand (RANKL) expression, which is also directly activated via the recruited extravasated neutrophils. RANKL drives the activation and maturation of osteoclast precursor to be an active osteoclast that predisposes to bone resorption. The recruited neutrophils have a tissue degradation effect through inducing the expression of matrix metalloproteinases (MMP) and cytotoxic substances such as reactive oxygen species (ROS). The microbial-innate-adaptive cell interactions demonstrate some of the main mechanisms involved in the continuity of inflammation if not resolved, leading to tissue destruction.

Neutrophils can also induce the expression of receptor activator of nuclear factor kappa-B ligand (RANKL) on the osteoclast’s membrane, promoting bone resorption [158]. In addition, neutrophils promote Th17 cells which represent an osteoclastogenic T-cell subset that has been linked to bone loss [159], as well as a B-cell activator that promotes antibody production, and possible chronicity of inflammation [160]. During the transition from gingivitis to the advanced periodontitis lesion, the antimicrobial peptides of neutrophils seem to be replaced by increased activity of Langerhans dendritic cells and γδ-T cells, which bridge the innate and adaptive host networks, secreting an array of pro-inflammatory cytokines such as IL-1, IL-6, IL-17, TNF-α and IL-23 (Figure 6).

The induced-endothelial expression of intercellular adhesion molecule (ICAM)-1 and selectin receptors by capillary activation-associated bacterial invasion promotes leukocyte transmigration and exudation [161]. This exists in parallel with DEL-1 reduction within the periodontitis lesion, enhancing leukocyte infiltration to the inflamed target site. Commensal bacteria, accessory and other key pathogens can engage with their host counterpart through host-TLR and other PRRs such as the interaction between bacterial LPS with LPS-binding protein (CD14) [151] and associated TLRs on the surfaces of neutrophils, monocytes, macrophages and mast cells. This may be antagonized through the antimicrobial action of IL-37 [151]. However, these antimicrobial peptides, in association with chronic inflammation may serve as danger-associated molecular patterns (DAMPs) that activate inflammasomes with neutrophils, releasing superoxide derivatives that are toxic not only against bacteria but to the host itself, resulting in extensive tissue damage. On the other hand, within the advanced periodontitis lesion, prostaglandin E2 production is enhanced and may activate pro-coagulant and pro-inflammatory host responses. It is worth noting that these reactions could potentially be reversed using pro-resolving lipid mediators such as resolvins [151].

The complement defense system comprises various proteins, mediators, effectors and complexes that exert antimicrobial defenses at the onset of acute inflammation/starting of gingivitis, and which become dysregulated/subverted at the advanced periodontitis stage. Firstly, GCF which carries complement proteins such as C3b, an opsonin, is secreted into the gingival crevice following transmigration through the junctional epithelium and augments the inflammatory response, enhancing phagocytosis and microbial killing by neutrophils [151]. As the inflammation proceeds, the dysbiosis-associated microbiota have the ability to uncouple the bactericidal activity of the inflammatory response from the inflammation itself, thereby exploiting it to their advantage [131]. Pathogen survival requires inflammation for nutrient supply, but the host response must also be evaded or modulated for bacterial survival.

**Figure 7.**
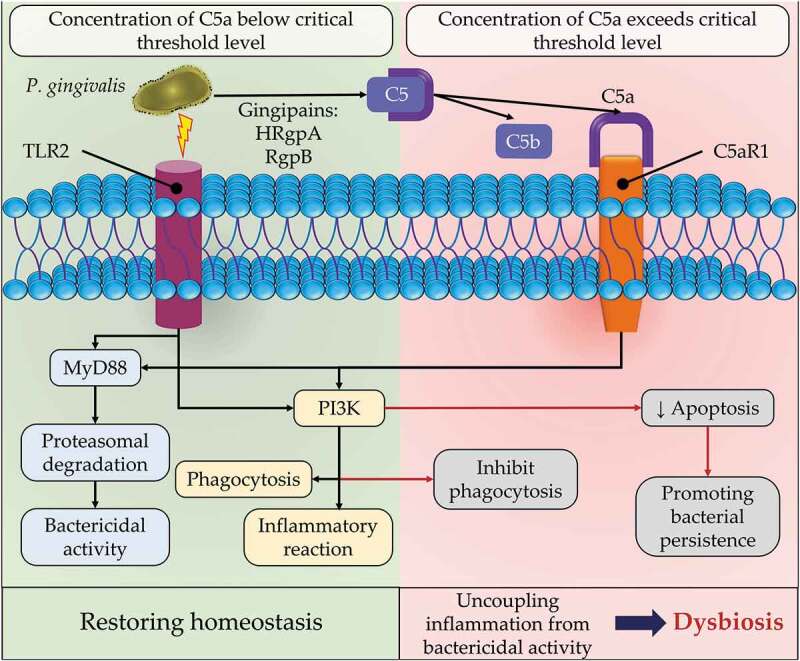
*Porphyromonas gingivalis* enhancing dysbiosis through uncoupling of inflammation from bactericidal activity of the phagocytic cells. *P. gingivalis* interacts with Toll-like receptor (TLR2), and acts on complement component 5 (C5) through *P. gingivalis*-associated arginine gingipains (HRgpA and RgpB) to produce C5a and C5b. C5a ligand then interacts with its specific complement C5a receptor (C5ar1) that together are co-activated with TLR2 on the surface of phagocytic cells. The cross-reactivity of both receptors could induce myeloid differentiation primary response 88 (MYD88)-induced inflammation or be blocked if MyD88 is inactivated. However, the same cross-reactivity of TLR2-C5aR1 complex could bypass MyD88 and induce the phosphoinositide 3-kinases (PI3K) pathway that may induce inflammation in phagocytic cells. In a similar manner, the activated PI3K could inhibit bacterial phagocytosis/apoptosis and supress phagolysosomal maturation, enhancing bacterial persistence. The latter mechanism is dependent on increased concentration of C5a beyond a threshold level (100 nM). The insurance of bacterial survival while inducing inflammation results in increased inflammophilic pathobionts and enhances dysbiosis.

For inflammophilic bacteria, the tactic of immunosuppression appears unsuitable as it creates an inflammation-free environment deprived of the essential nutrients required for bacterial colonization and persistence. As a principle of the keystone pathogen concept, *P. gingivalis* has the ability to impair the bactericidal properties of the host response, in particular PMNs, while still inducing inflammatory crosstalk [162–164], providing a significant benefit to the whole microbial community. In this situation, uncoupling bactericidal activity from inflammation may be achieved through complement, TLR signaling and cytokines manipulation by key pathogens such as *P. gingivalis* [140]. *P. gingivalis*–induced immune subversion has been investigated at the level of complement C5a receptor 1 (C5aR1) and TLR2 crosstalk signaling [164,165]. The downstream outcome of C5aR1-TLR2 could separate the protective pathway of the TLR2-Myeloid differentiation primary response 88 (MYD88) from TLR2-MyD88-Phosphoinositide 3-kinases (PI3K) pathway, inhibiting phagocytosis and enhancing inflammation [164]. Moreover, *P. gingivalis* may bypass MyD88, promoting anti-phagocytic/antiapoptotic TLR2-PI3K signaling, thereby suppressing phagolysosomal activity, leading to intracellular persistence of pathogens [166] (Figure 7). Notably, this evasion mechanism is related to the gingipains, HRgpA and RgpB, whose activity can upregulate levels of C5a (>100 nM), that in turn modulates upstream of the PI3K pathway [167]. The presence of TLR2 is crucial in *P. gingivalis*-induced immune subversion and inflammation regardless of the presence of MyD88, as shown in *P. gingivalis*-induced bone loss in mice [166]. Downstream of TLR2-C5aR1 and upstream of PI3K represent potential therapeutic targets that can be exploited to inhibit dysbiosis of the dental biofilm while maintaining a beneficial inflammatory response.

**Figure 8.**
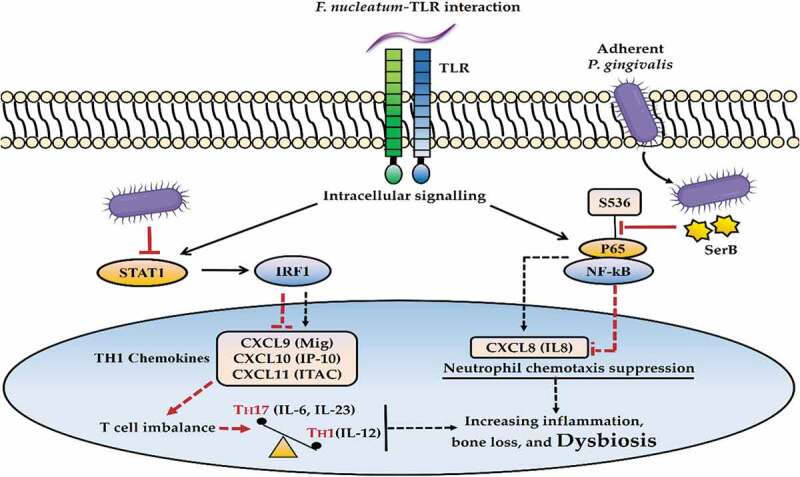
Porphyromonas gingivalis-induced chemokine paralysis. The activated Toll-like receptors (TLR), following interaction with oral pathobionts such as Fusobacterium nucleatum, induce proinflammatory signaling mechanisms. The invading keystone pathogen (P. gingivalis) can suppress interleukin (IL)-8 production through dephosphorylation of S536 residue of p65 subunit of nuclear factor kappa B (NF-kB) by the activity of serine phosphatase B (SerB), disrupting neutrophil recruitment. Similarly, the expression of chemokine CXCL9 (Mig), CXCL10 (IP-10), and CXCL11 (ITAC) could be inhibited through blocking the signal transducer and activator of transcription 1 (STAT1)-interferon regulatory factor 1 (IRF1) pathway by P. gingivalis, leading to T cell imbalance, including TH17 activation (IL-6, IL-23) and TH1 suppression (IL-12). These immune subversion mechanisms lead to enhanced inflammatory responses and dysbiosis.

The benefits for the whole microbial community are supported by another immune subversion mechanism induced by *P. gingivalis*, localized chemokine paralysis [140]. The suppression of chemotactic IL-8 is induced through the action of serine phosphatase B (SerB) whereby *P. gingivalis* dephosphorylates S536 residue of p65 sub unit of nuclear factor kappa B, suppressing transcription of CXCXL8 (IL-8) [168]. In addition, the chemokine suppression of CXCL9, CXCL10 and CXCL11 is mediated through the invading *P*. *gingivalis* blocking the signal transducer and activator of transcription 1 (STAT1)-interferon regulatory factor 1 (IRF1) pathway in epithelial cells and neutrophils. This promotes T cell imbalance by suppression of T_H_1-associated activities, including downregulating IL-12 and activation of T_H_17-associated activities including upregulation of pro-inflammatory IL-6 and IL-23 cytokines, thereby enhancing the inflammatory response and bone loss [169] (Figure 8).

The aforementioned examples of immune subversion/inducing inflammation help clarify the uncoupling bactericidal activity of inflammation by *P. gingivalis*. This is evident through chemokine paralysis and its debilitating influence on immune patrolling parallel to the buildup of a dysbiotic biofilm. Whereas, perturbation in the homeostatic relationship between protective and destructive immunity, induced by disturbance of T_H_1-induced chemokines [156], could enhance the non-symbiotic environmental alterations towards a well-characterized dysbiotic biofilm. From stable versus diseased sites, longitudinal meta-transcriptomic investigation of microbial communities has shown that *P. gingivalis* represents the only red complex bacterium that expresses a wide range of virulence factors in a proportionate manner from healthy sites, to be overexpressed with increasing inflammation and development of dysbiosis and disease progression. Meanwhile, other members such as *T. denticola* and *T. forsythia* have contributed their virulence as pathobionts at later stages of tissue breakdown, accelerating disease progression [126]. Thus, *P. gingivalis* could promote the fitness of the whole microbial ecology in favorable nutrient rich and disease-provoking inflammatory environments. However, the keystone pathogen hypothesis is a hypothesis, and whilst *P. gingivalis* is undoubtedly a key pathogen, it cannot be a keystone organism as it is only identified in 60% of cases of periodontitis, and biofilm survival is not dependent upon its presence.

Current knowledge about the shift of the periodontal microbiome from health to disease, together with the understanding of immune-inflammatory responses to biofilm accumulation provide novel opportunities to prevent and treat periodontal disease. One of these approaches is dedicated towards restoring symbiosis by using probiotics and prebiotics [170]. Several randomized clinical trials (RCTs) have evaluated the efficacy of using agents such as *Lactobacillus reuteri*, *L. rhamnosus* SP-1, *Bifidobacterium lactis*, and *S. oralis* in the treatment of gingivitis and periodontitis [171–175]. Despite the promising results observed with using probiotics in such trials, clinical outcomes expressed as clinical attachment gains, reduced bleeding scores, closure of periodontal pockets, and the composition of the biofilm are inconsistent. This has been attributed to heterogeneity in the mode of administration, frequency, dosage, and different treatment protocols across studies. Therefore, use of probiotics in the treatment of periodontitis is still not recommended by the latest S3-treatment guidelines [176]. However, a concrete conclusion cannot be drawn without conducting further highly standardized RCTs.

## Summary

The diversity of bacterial species and matrix macromolecules of the biofilm render the investigation of their exact role in the pathogenesis of periodontal disease challenging, requiring sophisticated multispecies biofilm models that are technically and financially demanding. Nevertheless, the available literature provides insights into, and understanding of the hosts’ immunological and inflammatory responses to the accumulation of the dental biofilm on tooth surfaces and within subjacent supra- and subgingival niches.

Indeed, colonization by resident microbiota, i.e. symbiotic biofilm formation, is evident during periodontal health. These bacteria are beneficial and prevent colonization of potentially pathogenic species. Once this homeostasis is compromised, dysbiosis leads to the development of periodontitis.

Whilst PMNs have a primarily protective role against periodontal infection, they are considered as major contributors to the exaggerated inflammation-associated tissue destruction. This notion is supported by the abundance of these cells through different stages of periodontitis, starting with gingivitis to early periodontitis and episodes of acute exacerbation of periodontitis. The tissue breakdown is further mediated by other components of the immune/inflammatory system including Ig, complement proteins, and the cross-talk between proinflammatory and regulatory cytokines. Later, specific T cell subsets, e.g. γδ-T and TH1and TH17 cells bridge the innate and adaptive immune response and sustain the chronicity of inflammation, leading to increased local oxidative stress responsible for further damage to the periodontal tissues.

The dysbiosis-associated pathogen *P. gingivalis* exhibits a large arsenal of virulence factors that in presence of nutrients are essential for colonization and persistence, immune system evasion, impairment of inflammatory cell function and induction of immune subversion and consequent inflammation. These events pave the way for overgrowth of inflammophilic pathobionts and provide protection for *P. gingivalis* from the inflammatory response. The latter is considered as a key pathogen that is available in relatively low-abundance and cannot induce tissue destruction or bone loss by itself, but achieves this by manipulating other commensals and modulating the host response, subsequently disrupting host homeostasis and inducing dysbiosis. Future treatment strategies could exploit the growing knowledge of host/bacterial and interbacterial interactions to prevent periodontal dysbiosis and treat periodontitis. However, further in-depth investigations are necessary to gain better understanding of these interactions and determine the exact role of each pathogen in the formation and development of a dysbiotic biofilm.
